# The Use of Computed Tomography Perfusion on Admission to Predict Outcomes in Surgical and Nonsurgical Traumatic Brain Injury Patients

**DOI:** 10.7759/cureus.5077

**Published:** 2019-07-04

**Authors:** Tye Patchana, Ryan Dorkoski, Bailey Zampella, James G Wiginton, Raed B Sweiss, Rosalinda Menoni, Dan E Miulli

**Affiliations:** 1 Neurosurgery, Riverside University Health System Medical Center, Moreno Valley, USA; 2 Environmental and Plant Science, Ohio University, Athens, USA

**Keywords:** traumatic brain injury (tbi), ct perfusion, brain injury, cerebral blood, neurotrauma

## Abstract

Introduction: The objective of this study was to investigate if data obtained from a computed tomography (CT) perfusion study on admission could correlate to outcomes for the patient, including the patient’s length of stay in the hospital and their initial and final Glasgow Coma Scale (GCS), as well as the modified Rankin Scale (mRS) on discharge. We present an initial subset of patients fulfilling the inclusion criteria: over the age of 18 with mild, moderate, or severe traumatic brain injury (TBI). Patients admitted with a diagnosis of TBI had CT perfusion studies performed within 48 hours of admission. GCS, length of stay, mRS, and discharge location were tracked, along with the patient’s course of hospitalization. Initial results and discussion on the utility of CT perfusion for predicting outcomes are presented.

Methods: Patients exhibiting mild, moderate, or severe TBI were assessed using CT perfusion within 48 hours of admission from January to July 2019 at the Arrowhead Regional Medical Center (ARMC). The neurosurgery census and patient records were assessed for progression of outcomes. Data obtained from the perfusion scans were correlated to patient outcomes to evaluate the utility of CT perfusion in predicting outcomes in surgical and nonsurgical TBI patients.

Results: Preliminary data were obtained on six patients exhibiting TBI, ranging from mild to severe. The mean GCS of our patient cohort on admission was eight, with the most common mechanism of injury found to be falls (50%) and motor vehicle accidents (50%). Cerebral blood volume (CBV) seemed to increase with Rankin value (Pearson's correlations coefficient = 0.43 but was statistically insignificant (P = 0.21)). Cerebral blood flow (CBF) was found to be correlated with CBV, and both increased with Rankin score (Pearson's correlation coefficient = 0.56) but were statistically insignificant (P = 0.27). These results suggest that with a larger sample size, CBV and CBF may be correlated to patient outcome.

Conclusion: Although more data is needed, preliminary results suggest that with larger patient populations, CT perfusion may provide information that can be correlated clinically to patient outcomes. This study shows that CBF and CBV may serve as useful indicators for prognostication of TBI patients.

## Introduction

It has long been known that the cornerstone of treatment for non-surgical traumatic brain injuries is medical management following the primary brain injury. Focus has shifted to prevention of secondary brain injury and toward the hours, days, weeks, and months afterward. Traumatic brain injury (TBI) causes a high burden on society as it affects many young patients in their twenties and thirties - their most productive occupational years. Several modalities exist in the evaluation of TBI, including initial computed tomography (CT) scans of the head, magnetic resonance imaging (MRI) sequences, CT angiography, magnetic resonance angiography (MRA), and magnetic resonance venography (MRV) studies. One modality that has gained interest in the evaluation of acute stroke is CT perfusion (CTP). The traditional use of CTP studies has occurred in the evaluation of acute vascular insufficiency and ischemia that may be rescued by neuro-interventional procedures, but it has also been studied in the application of brain death determination and TBI [1]. Recent studies have found that CTP can be a useful adjunct to traditional CT, resulting in changes in clinical management [2]. The potential use of CTP as a clinical indicator depends on its ability to delineate changes in cerebral blood flow (CBF), cerebral perfusion pressure, and ultimately, ischemic insults to the brain. Because measurement of the pressure in intracerebral arteries is not practical, neurosurgical intensivists must rely on secondary and indirect metrics to evaluate the flow of blood within the cranium. The result of decreased cerebral perfusion and subsequent ischemia are manifold - the accumulation of lactic acid secondary to anaerobic glycolysis, increased membrane permeability (as a result of depleted adenosine triphosphate (ATP)), and edema as the physiological mechanisms that govern CBF fails [3]. Indeed, ischemic signs have been found in over 90% of TBI patients upon autopsy [4-5]. Several studies have shown that focal or diffuse cerebral ischemia occurs following TBI, even in the very earliest stages [6-8]. Indeed, intracranial pressure (ICP) levels are followed as a surrogate for both herniation and ischemia, with a proven correlation to functional outcomes in TBI patients [9].

CTP is predicated upon acquisition of a contrast bolus passing through the cerebral vasculature and surrounding brain. It captures both the timing of arrival of the bolus (Tmax) and the timing of passage of the bolus (mean transit time (MTT)) [10]. Recently, its utility for the detection of lung cancer tumor blood flow, coronary angiography in heart transplant patients, and the hemodynamics in Moyamoya disease is being investigated [11-13]. To date, studies involving CTP have focused on the measurement of MTT, CBF, and cerebral blood volume (CBV). CBF has previously been found to be the most important perfusion parameter predicting the Glasgow Outcome Score (GOS) [14]. These measurements are centered around particular regions of interest (ROI) within the brain parenchyma. Further, it has been found that CTP can be correlated to PbrO2 in these ROIs [15-16]. At ICP levels greater than 20 mmHg, CBF and MTT have been found to have a negative and positive correlation, respectively [17].

The addition of CTP studies adds just five minutes in the appropriate clinical and radiological setting and are based on the addition of iodine-based contrast [18]. It is known that in acute phases of head TBI, there are hemodynamic changes present. CTP can help elucidate these changes and help to decide what, if any, predictive value they have for patients with TBI. Previous studies have shown that reduced CBF and CBV on admission were predictive of the outcome in severe head injury when compared to the GOS at three months [19]. Additionally, the evaluation of perfusion surrounding contusion may be evaluated by CTP studies [20].

With the development and initial application of new technology, there is always an adjustment period when new technology is tested and eventually implemented in a useful way. Our goal was to observe if the use of CTP in the TBI patient population can change it from an academic exercise to a useful tool. Additionally, we aim to investigate whether CTP scanning can become an aid in the management of TBI patients. One advantage of CTP over other perfusion studies (xenon-enhanced CT, single-photon emission computed tomography (SPECT), positron emission tomography (PET), perfusion CT, and magnetic resonance (MR)) is its availability in the emergency department (ED) and trauma centers. Additionally, it can be performed as an add-on to non-contrast CT of the head, the traditional imaging of choice for first-line imaging in TBI patients. The extra dose of contrast is minor compared to contrast administered to evaluate the aortic injury and its branches in the chest, abdomen, and pelvis that trauma patients get typically [21]. We hypothesize that CTP can be used to prognosticate outcome in patients presenting with TBI. Further, a correlation to GCS, mRS, and eventual discharge location can be made in a subset or all TBI patients.

## Materials and methods

CTP imaging was obtained on an Aquilion® ONE Dynamic Volume CT system (Toshiba America Medical Systems, Inc., Tustin, CA, USA). Under our hospital CTP protocol, 50 cc of iodine contrast was used for the initial CTP. Patients admitted to ARMC who were greater than 18 years of age with mild (GCS 13 - 15), moderate (GCS 9 - 12), or severe (GCS 3 - 8) TBI were included in this study. Baseline measurements included GCS score, vital signs, and pupillary response. The primary outcome measured was GCS at admission, GCS at three days, GCS at five days, GCS at seven days, and GCS at discharge. Secondary endpoints included GOS, mRS, and discharge classified as a skilled nursing facility, acute rehab, or home. The duration of the study included January to July 2019 and included six patients.

These patients obtained a CTP upon initial presentation to the ARMC, most within 24 hours but all within 48 hours. CTP included the variables of MTT, CBV, CBF, time to peak (TTP), and Delay. The primary outcome investigated included GCS at admission, on Days 3, 5, and 7, and on the discharge date, as well as an mRS at discharge. Additionally, the outcome at discharge included the place of discharge, including a skilled nursing facility, acute rehab facility, or home. The hospital length of stay was also assessed.

All statistical analysis was done using Microsoft® Excel (2016) with the Analysis ToolPak (Microsoft® Corp., Redmond, WA, USA). Pearson's correlation coefficient (r) was calculated to determine the strength of association between the variables under consideration. The regression function was used to calculate P-values (α = 0.05). 

## Results

Preliminary data were obtained on six patients exhibiting TBI, ranging from mild to severe. The mean GCS of our patient cohort on admission was 8, with the most common mechanism of injury found to be falls (50%) and motor vehicle accidents (50%). Half of the patients were intubated upon arrival for an inability to protect their airway. Discharge location for our patient population included a skilled nursing facility, acute rehabilitation centers, and home. Only one patient died within 30 days of admission. Four of our patients (~66%) underwent surgical intervention while admitted, the most common intervention being the placement of external ventricular drain (EVD) and brain tissue oxygen monitoring using the Licox® brain tissue oxygen monitoring system (Integra Lifesciences Corp., Saint Priest, France) in two patients. One patient underwent a decompressive craniectomy, and another underwent placement of a subdural drain. The mRS ranged from 1 to 6, with the average mRS at discharge being 3, consistent with a moderate disability but able to walk without assistance. MTT, TTP, and Delay were not found to have any correlation with any other variables and showed significant variation between scans. 

**Figure 1:**
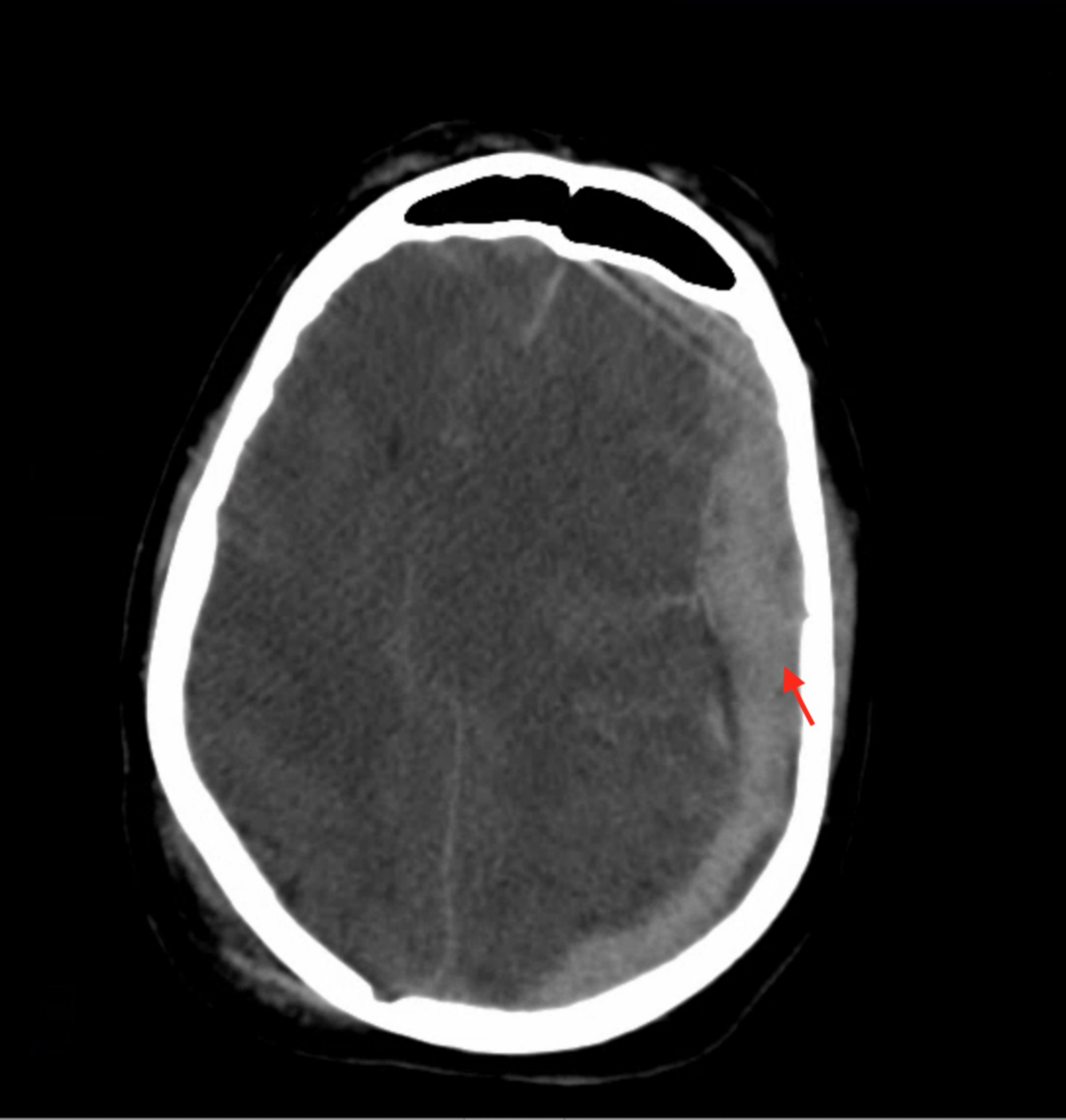
Computed tomography of the head demonstrating initial 22 mm traumatic subarachnoid hemorrhage with a 21 mm of midline shift

**Figure 2:**
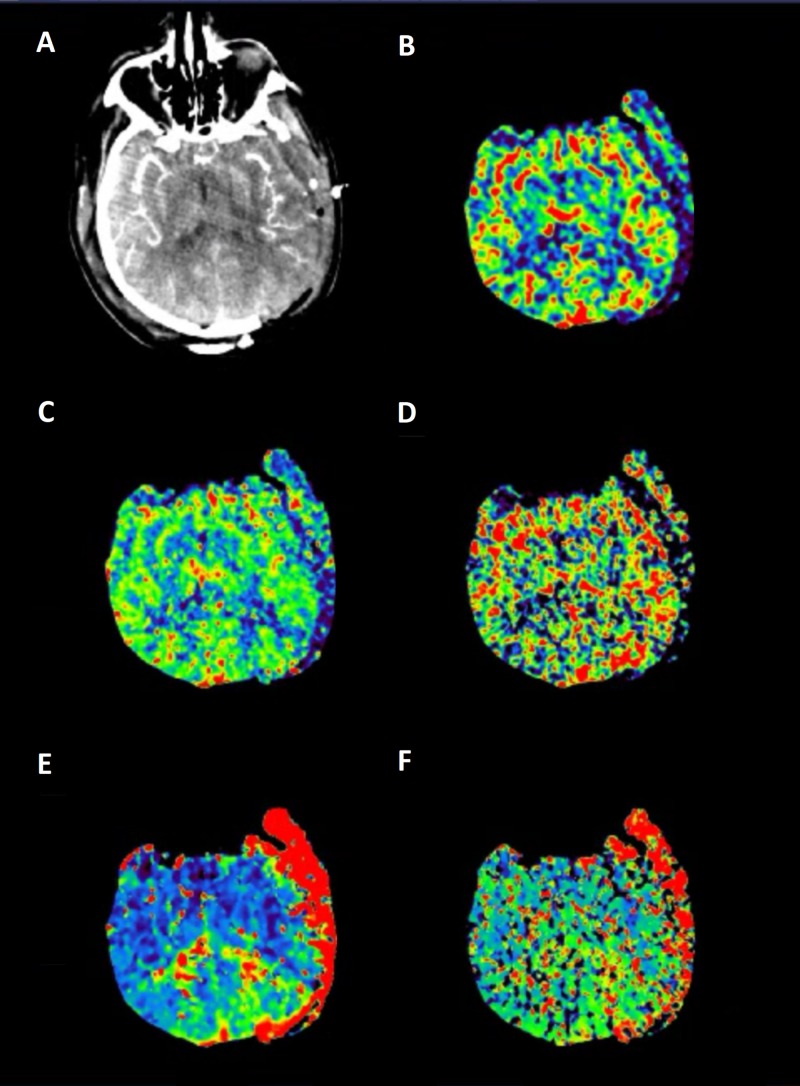
Computed tomography perfusion demonstrating (from left to right) (A) CTA, (B) CBV, (C) CBF, (D) MTT, (E) TTP, and (F) delay CBF: cerebral blood flow; CBV: cerebral blood volume; CTA: computed tomography angiogram; MTT: mean transit time; TTP: time to peak

**Figure 3:**
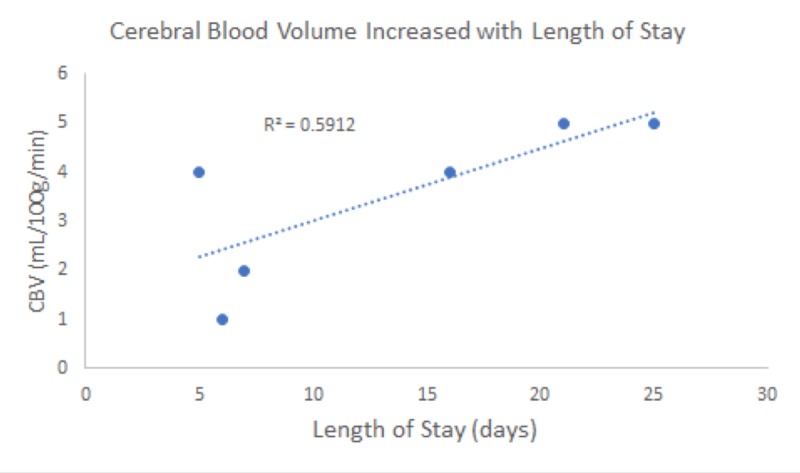
Cerebral blood volume (CBV) appeared to increase with the length of patient stay (P < 0.1). The 60% variation in CBV could likely be explained by the length of stay.

**Figure 4:**
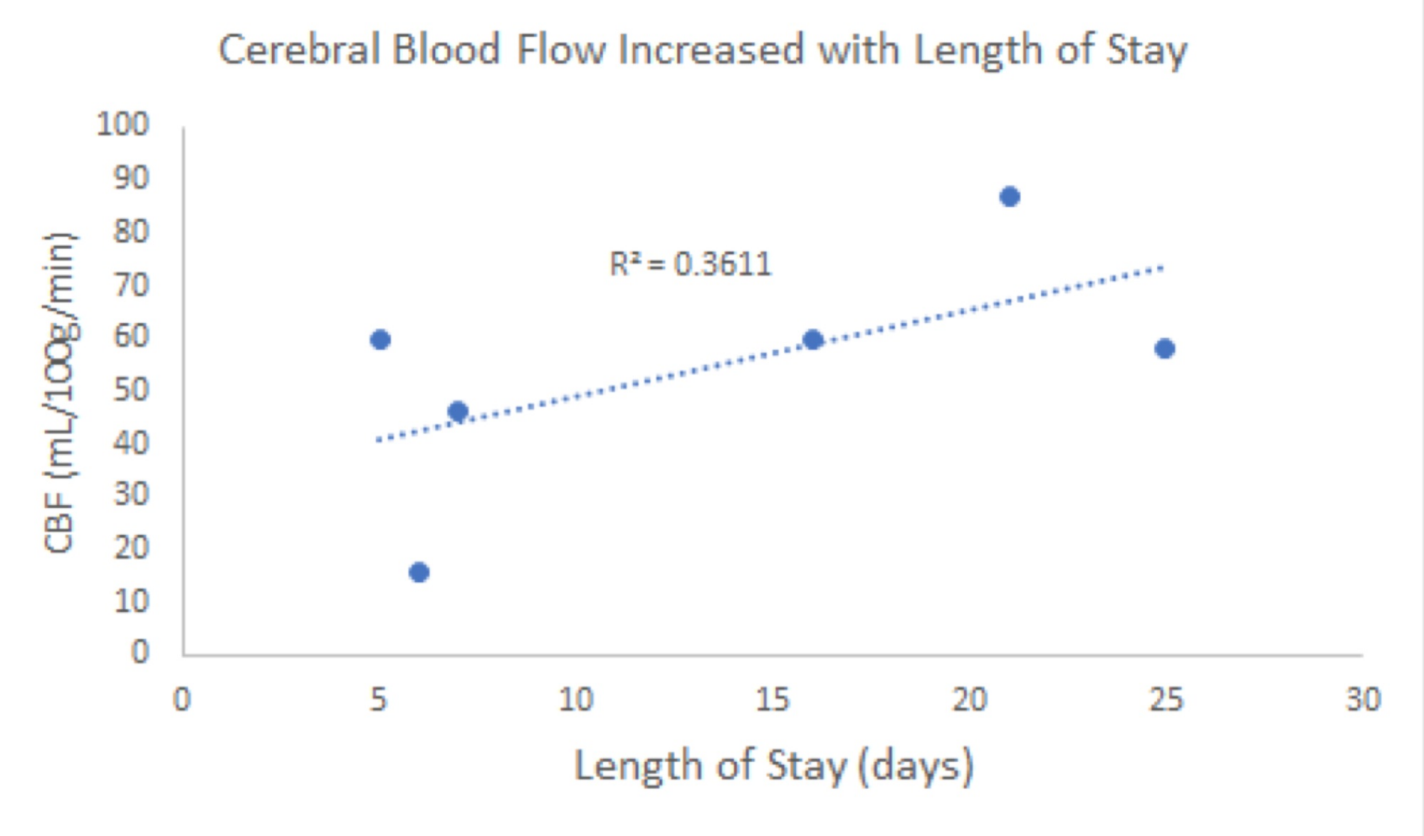
Cerebral blood flow (CBF) appeared to increase with the length of the patient stay (P < 0.1). The 36% variation in CBF could likely be explained by the length of stay.

**Figure 5:**
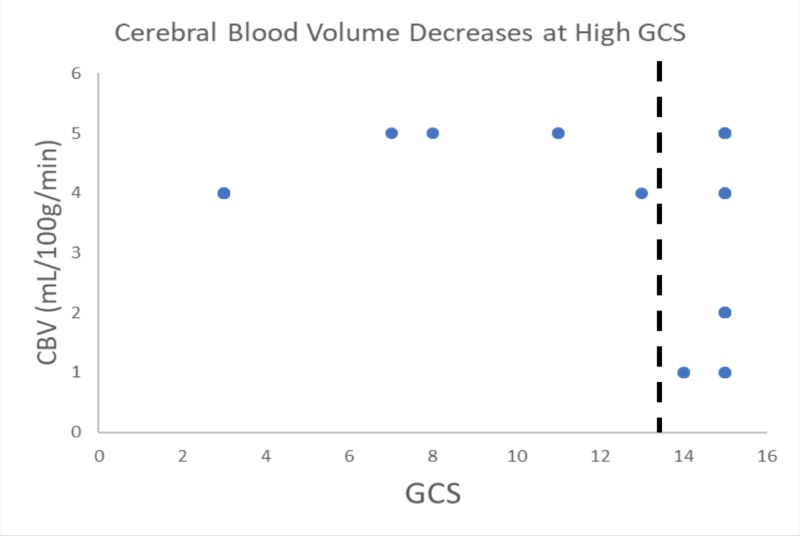
Cerebral blood volume (CBV) appears to drop at a Glasgow Coma Scale (GCS) threshold of approximately 14 The dashed line suggests possible GCS threshold. GCS scores of a patient taken at different time points were considered as separate data points. Visual overlap of points occurred frequently due to several data points being the exact same. This is data pattern exploration/observation and no statistical analysis was done here.

**Figure 6:**
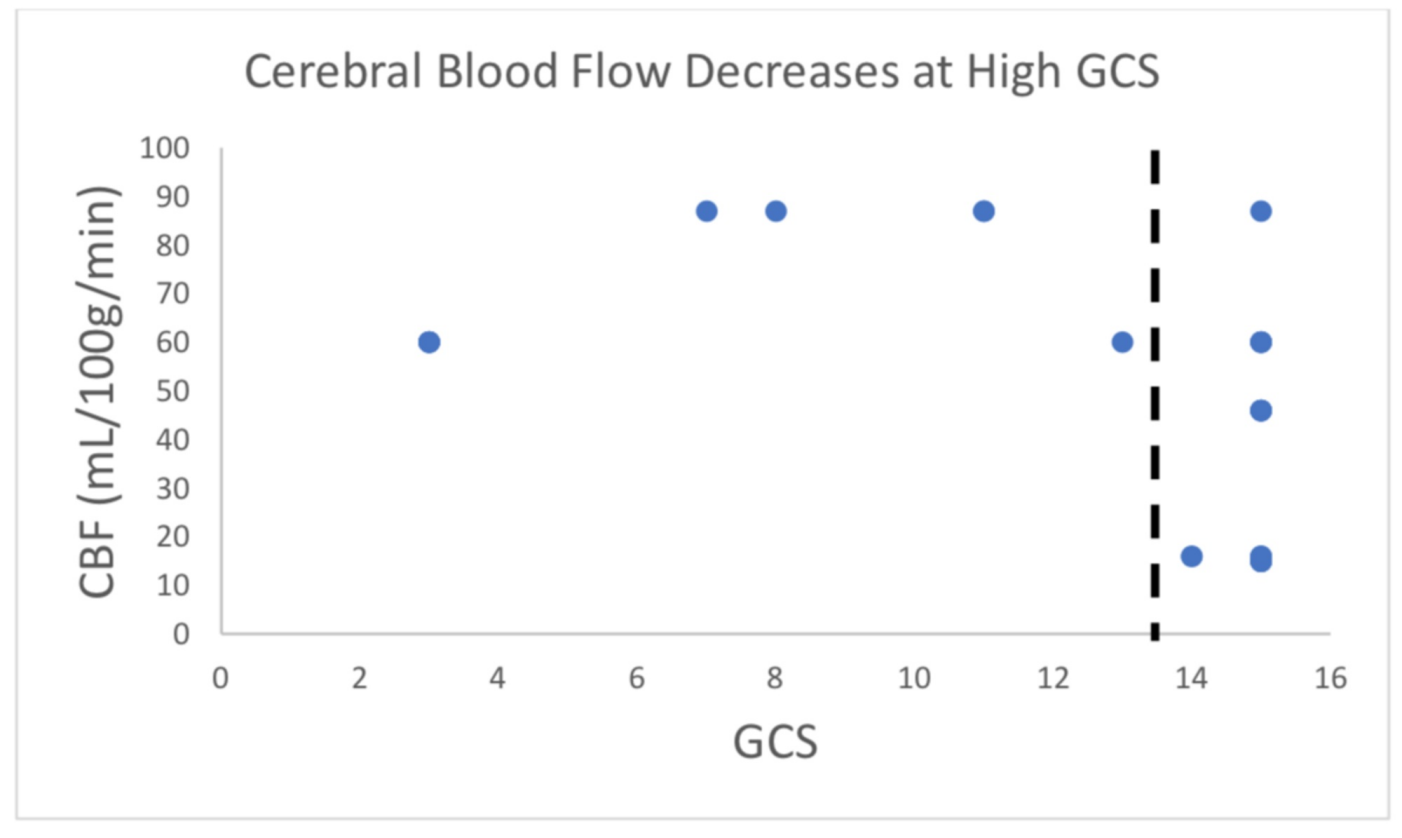
Cerebral blood flow (CBF) appears to drop at a Glasgow Coma Scale (GCS) threshold of approximately 14 The dashed line suggests possible GCS threshold. GCS scores of a patient taken at different time points were considered as separate data points. Visual overlap of points occurred frequently due to several data points being the exact same. This is data pattern exploration/observation and no statistical analysis was done here.

Our study included six patients presenting with various degrees of traumatic brain injury who received CTP studies within 48 hours of admission. Four (66%) of these patients went on to have surgical interventions performed. In this study, the patient received initial non-contrasted CT upon arrival as part of our hospital’s trauma protocol (Figure 1). If deemed a neurosurgical candidate, an intervention was offered and performed to stabilize patients. CTP studies were performed following the stabilization of the patients. Lesions were identified based on initial CT scans upon patient arrival. These lesions were then identified on CTP studies, and colorimetric data was used to estimate CBV, CBF, MTT, TTP, and Delay (Figure 2). This data was then correlated to tracked GCS, as well as mRS, during patient admission. Similar to previous studies [14], cerebral blood flow (CBF) was found to track with hospital length of stay. That is, those patients with longer hospital stays had a higher CBF calculated in the area immediately surrounding lesions on CTP (Figures 1-2). CBV and CBF, estimated in a similar manner, were also found to track with hospital length of stay in a similar manner (Figures 3-4). Finally, given that these variables correlated with the length of stay, we investigated if a relationship existed with GCS. Indeed, we found that both CBV and CBF appeared to drop at a GCS threshold of approximately 14 (Figures 5-6, respectively). MTT, TTP, and Delay were not found to have a correlation to hospital length of stay, GCS, or mRS. 

## Discussion

In this study involving patients admitted for TBI, CTP studies were used to assess MTT, cerebral perfusion pressure (CPP), CBV, and CBF. The classical division of TBI into primary and secondary insults has been known for some time, with the secondary insult being the target of intervention. Management of cerebral ischemia and intracranial hypertension, as a surrogate for cerebral perfusion pressure, fall within this domain. The main cause of death from TBI is from intracranial hypertension, acting as a surrogate for cerebral perfusion, which most patients with severe TBI experience [22].

It is known that when the pressure in the cranium begins to rise, venous blood is displaced through the jugular foramen and eventually the right atrium. This displacement is able to compensate for increased pressure for a time until mechanical compensation is overcome. As the cerebral arteries become compressed by increasing ICP, transmural pressure begins to dissipate, resulting in a decrease in CPP [23]. If neurosurgeons and neurointerventionalists can reliably identify who will be vegetative versus who will be high functioning, it would be an immense paradigm shift. Several attempts have been made to do just that [24].

This study included a cohort of six patients with traumatic brain injury who received CTP studies within 48 hours of admission after stabilization. Lesions were identified based on initial CT scans upon patient arrival. These lesions were then identified on CTP studies, and colorimetric data was used to estimate CBV, CBF, MTT, TPP, and Delay (Figure 6), then correlated to tracked GCS and mRS throughout the patient's hospital course. Similar to previous studies, CBF was found to track with hospital length of stay. However, those studies found that higher CBV/CBF correlated with higher GOS, though not with GCS, as our study examines [14]. 

Previously, CBF changes following TBI have been described as a triphasic pattern. In the acute phase of the first 12 hours, a 50% decrease in CBF has been found [6, 25]. After this, a rise in CBF to normal or higher values occurs and persists for several days [26]. CBF changes once again, with a fall in CBF remaining for up to several weeks. Altogether, these observations support the hypothesis that initial ischemia early in TBI is an important factor portending neurological outcome. Our study was designed to capture a snapshot of this initial pattern. As our study captured CBF/CBV in a relatively wide time frame of 48 hours, it is likely that values were captured at different points of the previously described triphasic pattern [6, 25-26]. Future studies may wish to further divide time frames into 12-hour increments for better discernment of changes of CBF/CBV. 

We found that patients with longer hospital stays had higher CBF calculated in the area immediately surrounding lesions on CTP (Figures 1, 6). CBV, estimated in a similar manner, was also found to track with hospital length of stay in a similar manner (Figure 2). Finally, given that these variables correlated with the length of stay, we investigated if a relationship existed with GCS. Both CBF and CBV appeared to drop at a GCS threshold of approximately 14 (Figures 3-4, respectively). MTT, TTP, and Delay were not found to have a correlation to hospital length of stay, GCS, or mRS, and thus were not represented graphically.

One limitation of this study is that patients can still progress neurologically and the results here only represent one snapshot in time. Neurological progress can be slow and involve years of physical rehabilitation. Indeed, studies on patient self-awareness, even five years post-TBI, have shown that patients are still learning to become self-aware of their neurological and cognitive deficits [27]. Further limitations include the fact that temperature, sedatives, and partial pressure of CO_2_ in arterial blood are known influencers of CBF and ICP [17]. CTP itself is also susceptible to motion artifact. This was observed in a small number of our patients undergoing CTP scans. Additionally, it is important to recognize that CTP provides a static measurement of CBF and CBV that may not account for changes in the hours and days following TBI. Importantly, the sample size of our patient pool is currently too small to provide statistically significant and conclusive results. However, this study provides some insight into the possible focus of future research involving CTP in the assessment of outcomes for neurosurgical and non-neurosurgical patients. 

## Conclusions

CT perfusion has the potential for providing at least a static representation of blood flow and metabolism within the brain in a noninvasive and quick manner. Importantly, a limitation of this study was that the sample size was too small to provide statistically significant and conclusive results. However, this lays the groundwork for future studies. This study hopes to add to the ability of neurosurgeons to better educate patients and their families on the expected clinical course following TBI. CT perfusion may be a future tool to aid in the prediction of patient outcome in traumatic brain injury. If neurosurgeons and neurointerventionalists can reliably identify who will be vegetative versus who will be high functioning, it would allow for better prognostication. 
